# Flexural Progressive Failure of Carbon/Glass Interlayer and Intralayer Hybrid Composites

**DOI:** 10.3390/ma11040619

**Published:** 2018-04-17

**Authors:** Qingtao Wang, Weili Wu, Zhili Gong, Wei Li

**Affiliations:** 1 College of Textiles, Donghua University, No. 2999, Northern Renmin Rd, Songjiang District, Shanghai 201620, China; a19870628wqt@126.com (Q.W.); 1152003@mail.dhu.edu.cn (W.W.); 2 Shanghai Starriver Bilingual School, No. 2588, Jindu Street, Minhang District, Shanghai 201100, China; 15026503803@163.com; 3 Key Lab of Textile Science & Technology, Ministry of Education, No. 2999, Northern Renmin Rd, Songjiang District, Shanghai 201620, China; 4 Center for Civil Aviation Composites, No. 2999, Northern Renmin Rd, Songjiang District, Shanghai 201620, China

**Keywords:** carbon/glass hybrid composites, interlayer hybrid, intralayer hybrid, flexural properties, progressive failure

## Abstract

The flexural progressive failure modes of carbon fiber and glass fiber (C/G) interlayer and intralayer hybrid composites were investigated in this work. Results showed that the bending failure modes for interlayer hybrid composites are determined by the layup structure. Besides, the bending failure is characterized by the compression failure of the upper layer, when carbon fiber tends to distribute in the upper layer, the interlayer hybrid composite fails early, the failure force is characterized by a multi-stage slightly fluctuating decline and the fracture area exhibits a diamond shape. While carbon fiber distributes in the middle or bottom layer, the failure time starts late, and the failure process exhibits one stage sharp force/stress drop, the fracture zone of glass fiber above the carbon layers presents an inverted trapezoid shape, while the fracture of glass fiber below the carbon layers exhibits an inverted triangular shape. With regards to the intralayer hybrid composites, the C/G hybrid ratio plays a dominating role in the bending failure which could be considered as the mixed failures of four structures. The bending failure of intralayer hybrid composites occurs in advance since carbon fiber are located in each layer; the failure process shows a multi-stage fluctuating decline, and the decline slows down as carbon fiber content increases, and the fracture sound release has the characteristics of a low intensity and high frequency for a long time. By contrast, as glass fiber content increases, the bending failure of intralayer composites is featured with a multi-stage cliff decline with a high amplitude and low frequency for a short-time fracture sound release.

## 1. Introduction

Fiber-reinforced composites are characterized by high specific strength and modulus, damage tolerance, fatigue resistance, and better designability than metals [1,2], which are extensively applied in industry and make a major contribution to the light-weighting products. However, there are some critical problems that we have to face, for instance, the cost of carbon fiber is high. In addition, the fracture strain is small, which may be a disadvantage for carbon reinforced composites subjected to the compression or flexural loading [3]. Although the tensile modulus of glass fiber is low, the fracture strain is high [4]. Therefore, it is proposed that a hybrid composite that embeds glass fiber into carbon fiber makes it possible to improve the failure strain of a composite and optimize the mechanical properties via the reasonable arrangement of hybrid ratios and layer structures [5,6].

The flexural properties enhancement is one of the important reasons for mixing carbon fiber into glass fiber, and many researchers have studied the effect on flexural properties and revealed some valuable findings. Sudarisman [7] found the flexural strength of interlayer hybrid composites could be improved obviously when glass fiber distributes in the upper surface. Additionally, an increase of glass fiber content makes the flexural strength and storage energy of interlayer hybrid composites present the positive hybrid effect. Dong [8] investigated the flexural properties of glass fiber and carbon fiber (C/G) hybrid composites, and found the modulus decreases as glass fiber content increases, and the flexural strength shows the positive hybrid effect, which was further proved by Kalantari [9]. Many researchers [10,11,12,13] studied the carbon/basalt hybrid forms, and found flexural properties greatly depend on the stacking sequences. Besides, Subagia [12] also revealed all hybrid composites present a positive hybrid effect. Flexural testing of carbon/SiC hybrid unidirection (UD) composites were conducted by Davies [14,15], a mixture of SiC fibers improves the flexural properties. Other hybrid forms like shape memory alloy/glass [16], carbon/Kevlar [17], polypropylene/glass [18], aramid/UHMPE [19], still attract some researchers. In recent years, since an awareness about green and environmental issue has increased, natural fiber hybrid composites like palm/glass [15], jute/carbon [20], glass/hemp/banana [21], jute/glass/carbon [22], glass/oil palm [23], poly(vinyl chloride)/wood flour/glass fiber [24], short banana/glass [25], glass/sisal [26], biofiber/glass [27], and jute/pineapple leaf/glass [28] have been reported extensively.

Besides investigations on the flexural properties and the hybrid effect, some work has been done on the flexural failure and theoretical contributions for hybrid composites. Ikbal [29] concluded that the failures subjected to bending loading include: compression failure, tensile failure and shear delamination. Dong [14,30] revealed the flexural failure is mainly dominated by the compressive failure. Giancaspro [31] investigated the flexural failure of woven glass/UD carbon hybrid composites, and found that while the specimens with one glass layer or with one or two carbon layers at the bottom face failed in tension, the compression failure always occurred even if specimens were reinforced at the bottom or upper surfaces, which was further demonstrated by Reis [32]. By locating the carbon fiber away from the neutral axis and at the neutral axis, the flexural property can be improved a lot [33]. Almansour [34] investigated the Mode II interlaminar fracture toughness of flax/basalt woven hybrid composites, and found the basalt fiber improved the interlaminar fracture toughness at the flexural crack initiation and propagation phase. Zhang [35] studied 3D woven flax/glass hybrid composites, where kink bands developed at the upper surface, and matrix cracking formed progressively at the tensile surface. In addition, Zhang [36] created a mesoscale model to simulate the flexural deformations and fractures via numerical analysis [37]. Sudarisman [4] used the optical microscopy to reveal the span-to-depth (s/d) exhibited a significant impact on the failure mechanisms, while the s/d = 16, the shear cracking existed at the neutral axis, whereas the s/d = 32 or s/d = 64 indicated the failure was featured with the kink bands and fiber buckling. Yet, Dong [38] found the s/d value has an influence on the stress-strain relationship, which would not change the failure modes. Huaguan [39] revealed the effective bending failure of GLARE laminates consists of the elastic stage, plastic stage, local fracture of fiber layer and delamination stage, and the failure of fiber leads to the delamination and further the complete fracture of a laminate. The hybridization effect on the flexural strength of carbon/glass hybrid composites was studied by Kalantari [9] to explain its mechanisms, and four classical lamination theories were employed to determine the stress distribution in a laminate and the methods for improving the flexural strength were given. Combined with the stress discontinuity of hybrid sandwich structures under bend loading, Fajrin [40] proposed the theoretical model under four-point bending. In [41], classical lamination theory models were adopted to predict elastic properties of jute-glass hybrid composites.

Interlayer and intralayer hybrid structures are the common hybrid forms, a majority of previous work mainly explains the flexural properties and the failure of interlayer hybrid, while little work has been conducted on intralayer hybrid structures. In our study, flexural properties of interlayer and intralayer composites with various hybrid ratios and layup structures were tested. Additionally, the bending behaviors and progressive failure modes were analyzed by combining with the light transmittance method and the fracture sound intensity-time curves.

## 2. Materials and Methods

### 2.1. Experimental Materials

**Figure 1:**
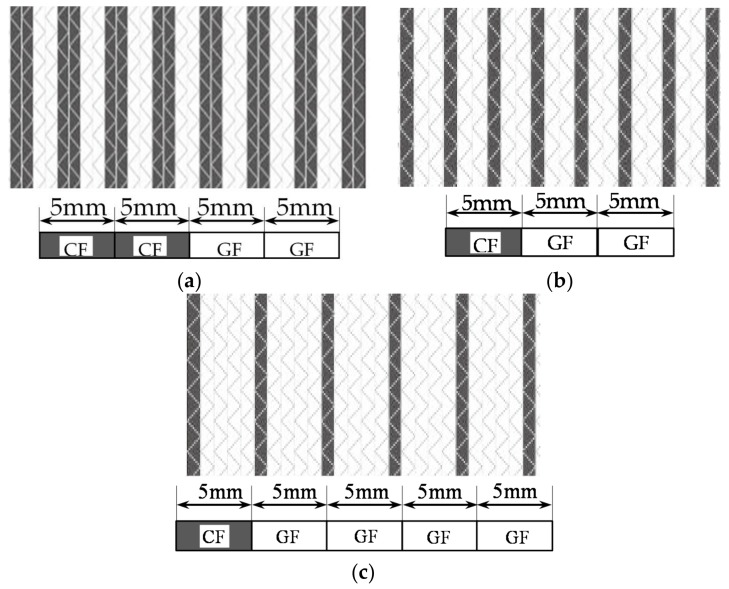
Schematic structures of three types of non-crimp fabrics (NCFs): (**a**) [C-G]; (**b**) [C-G-G]; (**c**) [C-G-G-G-G].

The CPIC ECT469L-2400 glass Fiber, TORAY T620SC-24K-50C carbon fiber and SWANCOR 2511-1A/BS epoxy resin were used in this research. Table 1 reports the mechanical parameters of raw materials, the specifications and fabric diagrams of non-crimp fabrics (NCFs), including a pure carbon fiber fabric, a glass fiber fabric and three kinds of C/G hybrid fabrics were presented in Table 2 and Figure 1, respectively.

### 2.2. Layer Structures Schemes of Interlayer and Intralayer Hybrid Composites

Interlayer hybrid structures were designed according to various stacking sequences of pure carbon and glass fiber fabric, as shown in Table 3. Intralayer hybrid structures were formed for the consideration of dislocation arrangements of carbon and glass fiber in various layers, which reflects the different dispersion degrees of composites, as presented in Table 4. The number indicates the dispersion degree, for example, for the structure [C-C-G-G]-0, the carbon and glass bundles are aligned with the upper and lower layers, when the structure [C-C-G-G]-0.5 indicates the fabric translates horizontally the half width of one bundle.

### 2.3. Experiments

**Figure 2:**
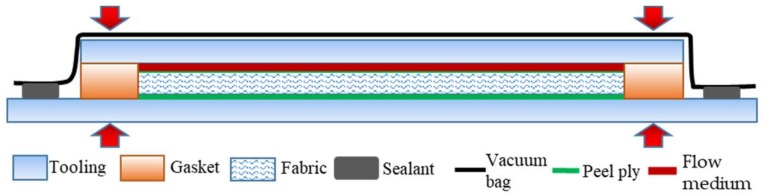
Schematic diagram of VARTM process.

Composites were prepared by the vacuum-assisted resin transfer molding process (VARTM), the schematic diagram of the process is shown in Figure 2. Gaskets with a height were used to support the upper glass mold and keep a constant thickness of the mold cavity, and the fiber volume fraction was kept at 50%. The flow medium/peel ply/fabric/peel ply were placed in the cavity from the top to the bottom, the whole mold was sealed with a vacuum bag, and G-shaped clamps were used to apply a force on the molds which fixed the thickness of the mold cavity and prevented the fabric from springing up. Then, air was evacuated by a vacuum pump, the resin was infused into the fabric. The curing condition was 120 °C for 8 h.

Five specimens of each laminate were tested, the three-point bending was performed at 1 mm/min speed. The specimen width of interlayer hybrid composites was set to 13 mm according to ASTM D7264, however, the specimen width of intralayer hybrid composites was established at the width of one unit cell, the cutting diagram was referring to [42], and carbon and glass bundles in specimens were symmetrically distributed. The support span-to-thickness ratio was established at 20. Due to differences of failure speeds and fracture modes between carbon and glass fiber, 50% force attenuation rate was given for the testing end parameter.

**Figure 3:**
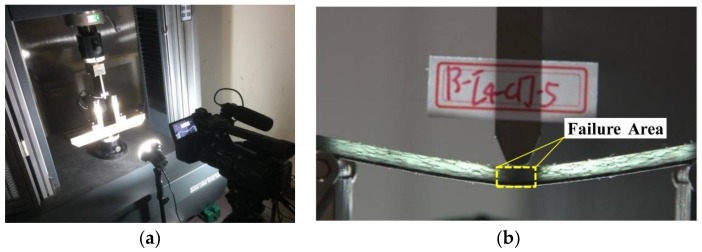
Three-point bending testing. (**a**) Light transmittance method; (**b**) Failure zone.

The universal testing machine was adopted to apply the bending load, the testing process was recorded by the camera. A beam of light was projected on the testing specimen, as shown in Figure 3a, and the failure zone of glass fiber was observed mainly due to the optical property of glass fiber epoxy composites. Since the glass fiber composites are transparent within light, after the failure occurs, the failure part contains air which makes the damage area appear opaque, as shown in Figure 3b. In addition, the sound of the flexural fracture process was recorded for analyzing the sound release of progressive failure.

## 3. Results and Discussion

### 3.1. Flexural Failure Modes of Carbon and Glass Fiber Composites

**Figure 4:**
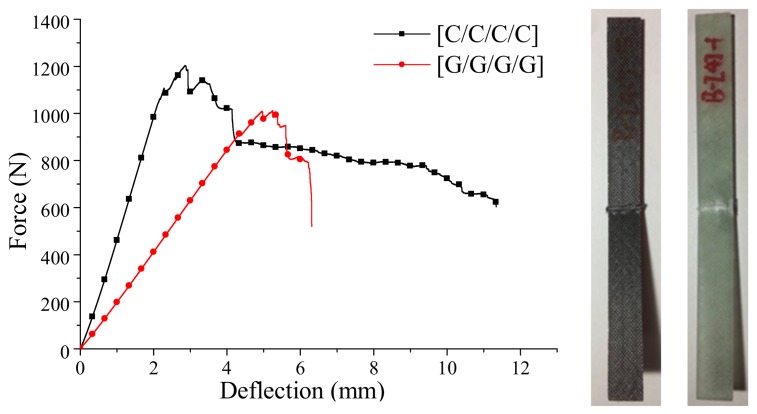
Force-deflection curves of carbon and glass fiber composites.

**Figure 5:**
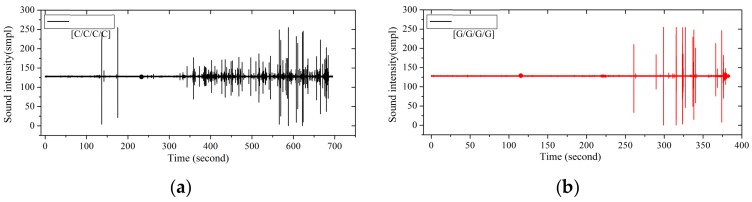
Flexure fracture sound intensity-time curve of carbon and glass fiber composites. (**a**) Carbon fiber composites; (**b**) Glass fiber composites.

The bending force-deflection curves for the pure carbon and glass fiber composites are presented in Figure 4, while the fracture sound during the bending failure is shown in Figure 5.

As seen from Figure 4, the bending force of carbon fiber composites shows a slight cliff-type decline at the beginning of failure, and then it falls slowly until the sample fails, which can be further demonstrated in the time-domain analysis in Figure 5a. The composite begins to fail in 130 s, after a two-stage high-intensity sound release, and the process appears mild and durable. In terms of the glass fiber composites, a more drastic cliff-type decline of the bending force appears during the failure process, and the glass fiber composite begins to fail in 260 s, and a high-intensity fracture sound releases several times before the failure.

**Figure 6:**
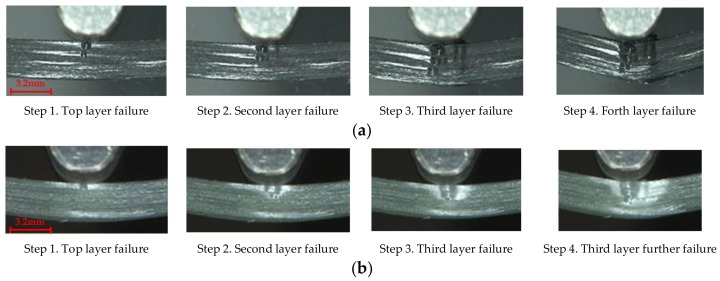
Flexure failure process of carbon and glass fiber composites. (**a**) [C/C/C/C]; (**b**) [G/G/G/G].

Figure 6a,b presents the bending progressive fracture process of the carbon and glass fiber composites, which is featured with the compressive failure. Subjected to bending load, the upper layers of composites are compressed, and the bottom layers are stretched. Since composites have the stronger tensile strength than the compressive strength [43], as composites reach the compressive strength, the compressive failures and buckling deformations in the top layers occur, firstly. Followed by the neutral plane moving down, the fiber cracking expands from the upper layers to the lower layers, then the fracture of lower layers occurs, and the failure area exhibits an inverted triangular shape.

**Figure 7:**
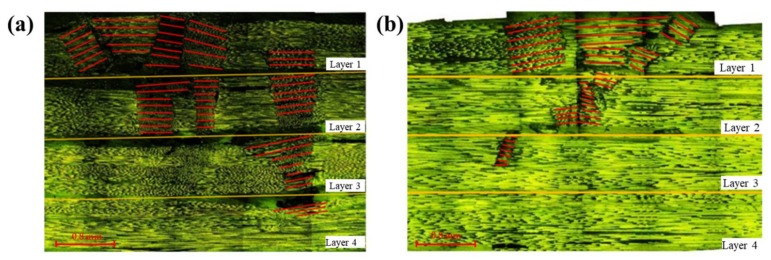
Microscopic fracture cross sections of (**a**) Carbon fiber composites; and (**b**) Glass fiber composites. Note: The red lines indicate fiber orientations in the failure zone.

The microscopic cross sections of flexural failure for carbon and glass composites are shown in Figure 7, in which, the red lines indicate fiber orientations after the bending failure. Fibers distribute along the 0 degree originally, after applied the loading, the failure is featured with the compression failure that distorts the fiber directions. The fracture zone for carbon fiber composites penetrates into the bottom layer to some degree, and it is larger than that of glass fiber composites in which the fracture only reaches to a small part of the third layer. Due to the high modulus and the low fracture strain of carbon fiber, once the loading reaches the fracture strain of carbon fiber, the upper layer fails. As the loading continues increasing, the remaining part still assumes the force, the fracture zone propagates to the lower layers gradually, which leads to the relatively slow trend of the failure process. The sound release process lasts for a long time, resulting in a large compression damage area in Figure 7a. By contrast, the damage strain of glass fiber is high, and a large displacement of the loading nose reserves a high energy for the specimen. As the deflection reaches the fracture strain of glass fiber, the specimen crushes sharply, and the failure ends in a short time, thus contributing to a small fracture zone, as shown in Figure 7b.

**Figure 8:**
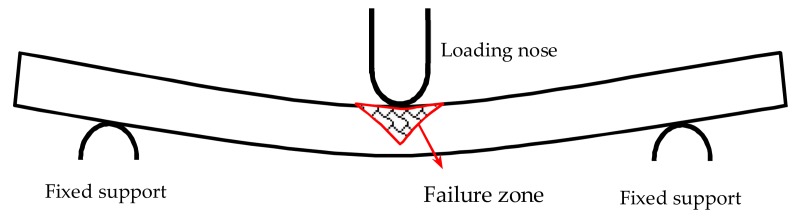
Flexure failure mechanism of composites.

The failure areas of pure carbon and glass fiber composites both exhibit an inverted triangular shape, the compressive stress decreases gradually from the upper layers to the lower layers causing a large compressive failure zone for the top layers. As a result, an inverted triangular failure shape appears in Figure 8.

### 3.2. Flexural Progressive Failure Modes of Interlayer Hybrid Composites

The flexural properties and bending progressive failures of interlayer hybrid composites were analyzed by mean of the light transmittance method and the fracture sound time-domain analysis in this part.

**Figure 9:**
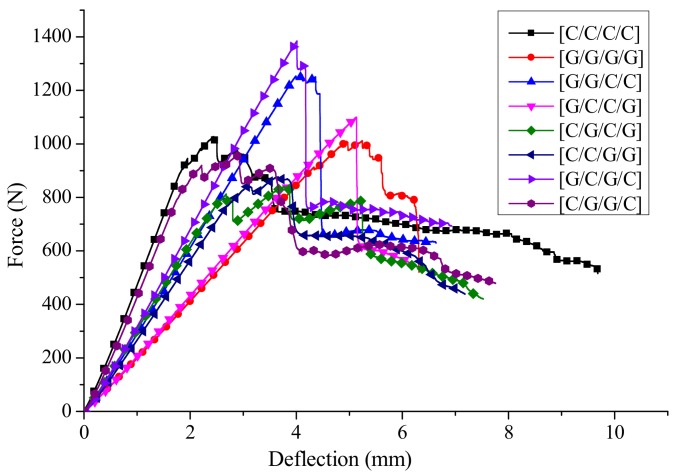
Flexural force-deflection curves of different interlayer stacking sequences with C/G = 1:1.

**Figure 10:**
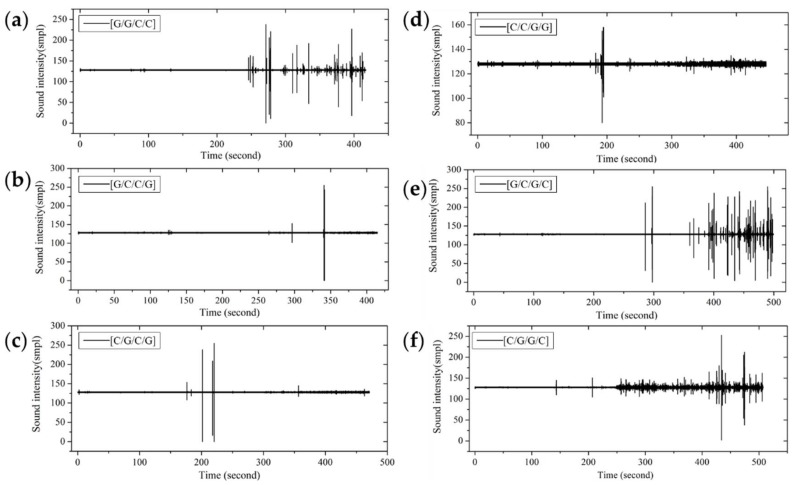
Flexure fracture sound intensity-time curves of various interlayer stacking sequences with C/G = 1:1: (**a**) [G/G/C/C]; (**b**) [G/C/C/G]; (**c**) [C/G/C/G]; (**d**) [C/C/G/G]; (**e**) [G/C/G/C]; (**f**) [C/G/G/C].

The deflection-force curves of interlayer hybrid composites with various layer structures for C/G = 1:1 are shown in Figure 9. Compared with the pure carbon or glass composites, the force attenuation with carbon fiber distributed at the upper surface, such as [C/G/C/G], [C/C/G/G] and [C/G/G/C], present a multi-stage slightly fluctuating decline as the failure starts, However, the force with glass fiber distributed at the upper surface only shows a one stage sharp cliff decline, then the force decreases slowly until the failure ends, like the structures [G/G/C/C], [G/C/C/G], [G/C/G/C]. These results can be further demonstrated by the time-domain curves in Figure 10.

From the time-domain curves of fracture sound intensity in Figure 10, the sound releases of interlayer hybrid composites with carbon fiber that distributes in the upper layers start early, due to the high compression modulus and the low fracture strain of carbon fiber. At the beginning of failure, there are several sound releases for high frequency, and then the releases present an extremely low frequency for a long time after a multi-stage failure. On the contrary, the failure start time with glass fiber that distributes in the upper layers is late, resulting from the high fracture strain of glass fiber, and there are only several releases until the failure ends.

**Figure 11:**
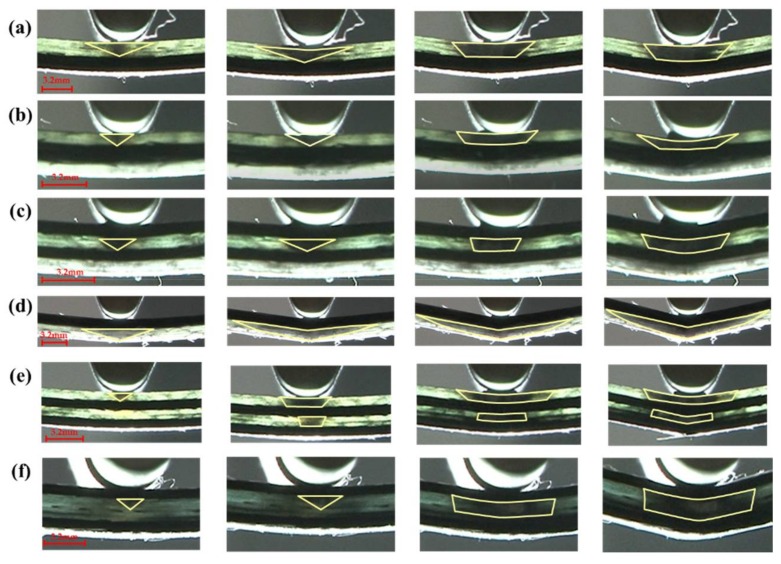
Flexure failure process of different interlayer stacking sequences with C/G = 1:1: (**a**) [G/G/C/C]; (**b**) [G/C/C/G]; (**c**) [C/G/C/G]; (**d**) [C/C/G/G]; (**e**) [G/C/G/C]; (**f**) [C/G/G/C]. Note: the yellow zone indicates the failure zone of glass fiber.

The flexural fracture process of interlayer composites with C/G = 1:1 is shown in Figure 11. The failure zone of glass fiber is an inverted triangular shape as the failure starts, with a larger deflection of the loading nose applied on the specimen, and various failure shapes for different layup structures appear. With carbon fiber distributed in the top layer, the fracture of the glass fiber below the carbon fiber propagates to the two sides from the center line, which looks like a diamond shape, as shown in Figure 11d. However, the interlayer hybrid composites with glass fiber distributed in the upper layers present an inverted trapezoid shape, as shown in Figure 11a,b,e. When there is a structure of carbon fiber sandwiching glass fiber, the fracture of glass fiber between the carbon layers exhibits an inverted trapezoid shape, such as Figure 11c,e,f.

**Figure 12:**
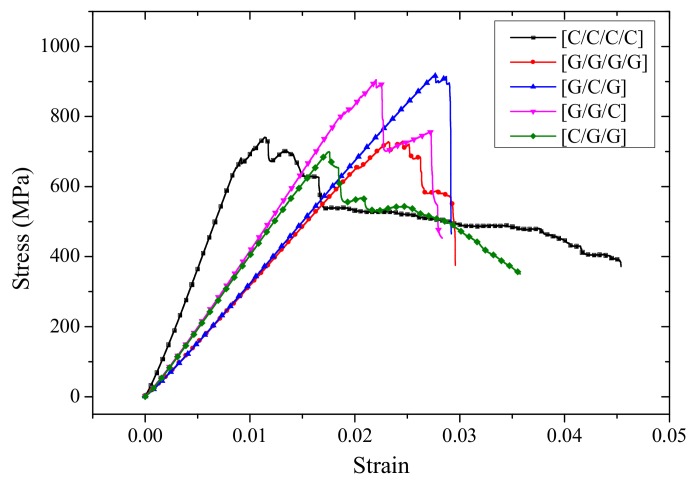
Stress-strain curves of different interlayer stacking sequences with C/G = 1:2.

Since the structure for C/G = 1:2 contain three layers while pure glass and carbon composites contain four layers, the force-deflection curves have been converted to stress-strain curves, which are shown in Figure 12. With glass fiber distributed in the upper layers, like [G/C/G] and [G/G/C], the stress-strain curves present a two-stage cliff decline before specimens fail. With carbon fiber placed in the upper layer, such as [C/G/G], the bending stress presents a multi-stage slight decline.

**Figure 13:**
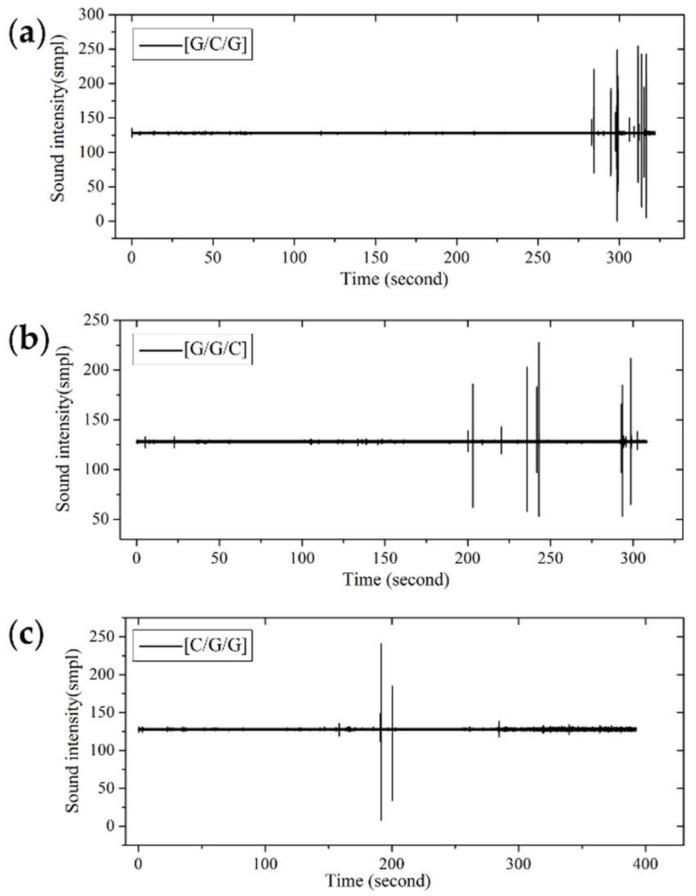
Flexure fracture sound intensity-time curve of various interlayer stacking sequences by C/G = 1:2: (**a**) [G/C/G]; (**b**) [G/G/C]; (**c**) [C/G/G].

In Figure 13, the failure start point of interlayer hybrid composites with the same hybrid ratio is determined by the layer sequence. With carbon fiber distributed in the top layer, such as [C/G/G], the failure time is early, while the failure time with carbon fiber distributed in the middle layer is late, since the low modulus and the high fracture strain of glass fiber distributes in the top and bottom layers, which delays the flexural failure. The failure time of hybrid structures with carbon fiber distributed in the bottom layer falls between that of these two structures.

**Figure 14:**
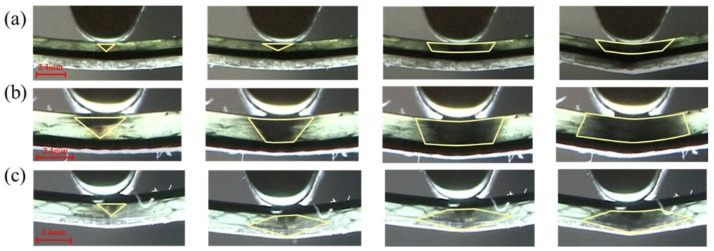
Flexure failure process of various interlayer stacking sequences of C/G = 1:2: (**a**) [G/C/G]; (**b**) [G/G/C]; (**c**) [C/G/G]. Note: the yellow zone indicates the failure zone of glass fiber.

Figure 14 shows the flexural failure process for C:G = 1:2, with the carbon fiber distribution in the top layer, such as [C/G/G], the failure area exhibits a diamond shape shown in Figure 14c due to the interlaminar shear between the glass fiber and carbon fiber, which prevents the compression failure of the glass fiber layers which are adjacent to carbon fiber. When carbon fiber is located in the middle layer, the failure mode of [G/C/G] is similar with that of structure [G/G/C], where the failure area is an inverted triangular shape at the start of failure, after the specimen is completely destroyed, and a trapezoid shape appears since the compressive crack propagates along the interface between the carbon and glass layers, as shown in Figure 14a,b.

**Figure 15:**
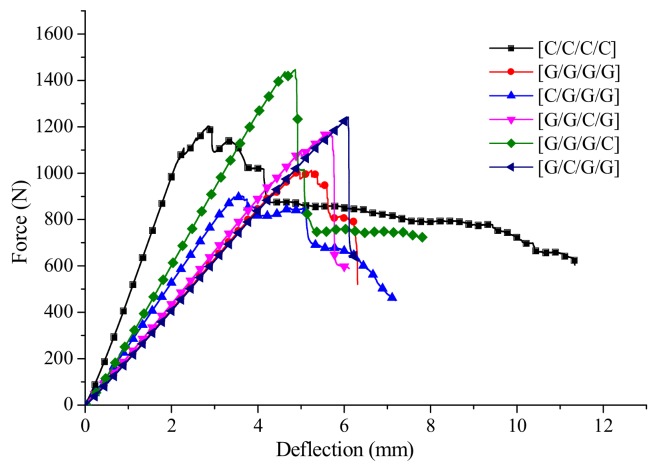
Force-deflection curves of various interlayer stacking sequences with C/G = 1:3.

The deflection-force curves of interlayer hybrid composites with C/G = 1:3 are shown in Figure 15. With carbon fiber distributed in the top layer, like [C/G/G/G], the force is characterized by a multi-stage fluctuating decline at the beginning of failure, and then decreases gradually. However, the failure modes of other structures show a one stage cliff-type decline before the failure.

**Figure 16:**
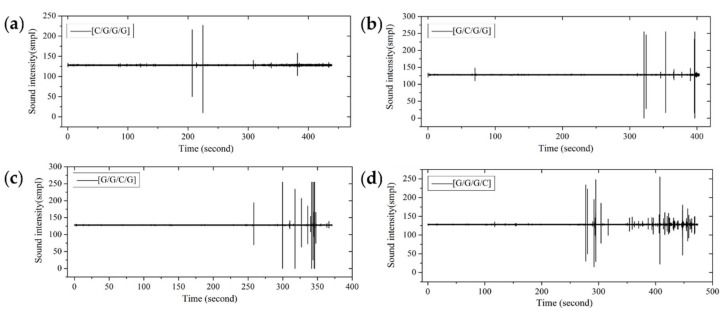
Flexure fracture sound intensity-time curve of various interlayer stacking sequences with C/G = 1:3: (**a**) [C/G/G/G]; (**b**) [G/G/C/G]; (**c**) [G/G/G/C]; (**d**) [G/C/G/G].

Figure 16 shows the fracture sound time-domain of interlayer hybrid composites for C/G = 1:3. With carbon fiber distributed in the top layer, the failure start time is early resulting from the high compression modulus and the low compression fracture strain of carbon fiber. By contrast, the failure start time of other interlayer hybrid structures, such as [G/C/G/G], [G/G/C/G], and [G/G/G/C], is late, due to the low compression modulus and the high compression fracture strain of glass fiber.

**Figure 17:**
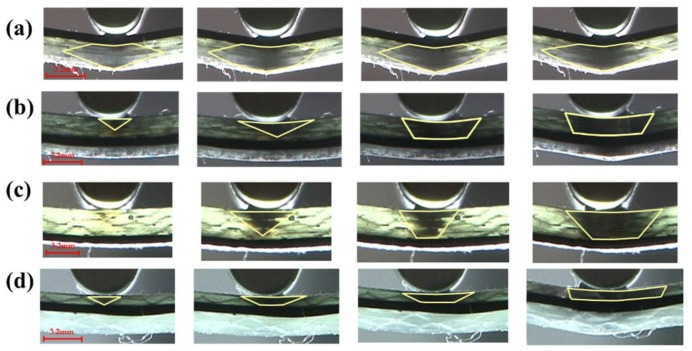
Flexure failure process of various interlayer stacking sequences with C/G = 1:3: (**a**) [C/G/G/G]; (**b**) [G/G/C/G]; (**c**) [G/G/G/C]; (**d**) [G/C/G/G]. Note: the yellow zone indicates the failure zone of glass fiber.

The flexure failure process for C/G = 1:3 are shown in Figure 17. It is concluded that the failure zone of glass fiber for interlayer hybrid composites with carbon fiber distributed on the top layer, such as [C/G/G/G], presents a diamond shape, shown in Figure 17a, since the shear stress between the top carbon layer and the glass layer stops the compression failure for the upper glass layer. When there is no carbon fiber in the top layer, the failure area shows an inverted trapezoid shape, as shown in Figure 17b–d.

**Figure 18:**
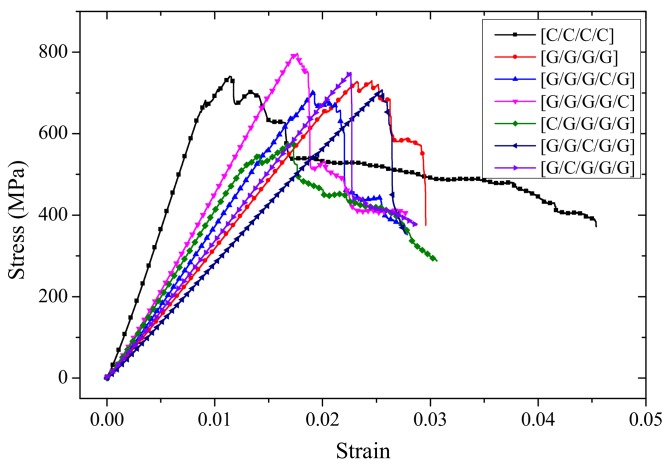
Stress-strain curves of various interlayer stacking sequences with C/G = 1:4.

**Figure 19:**
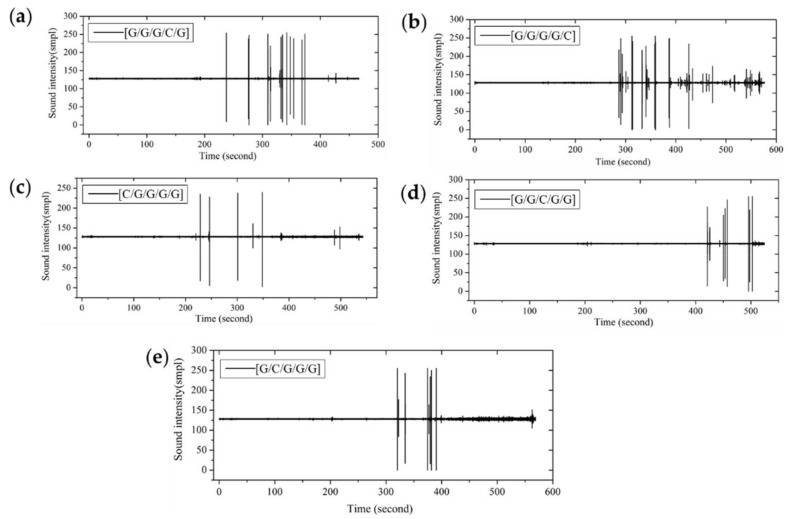
Flexure fracture sound intensity-time curve of various interlayer stacking sequences with C/G = 1:4: (**a**) [G/G/G/C/G]; (**b**) [G/G/G/G/C]; (**c**) [C/G/G/G/G]; (**d**) [G/G/C/G/G]; (**e**) [G/C/G/G/G].

For interlayer hybrid composites with C/G = 1:4, the laminates contain five layers, the stress-strain curves were used as shown in Figure 18. The failure force of [C/G/G/G/G] presents a slight multi-stage fluctuating decline as the premature failure of carbon fiber layer occurs. Besides, it leads to the failure time in advance as shown in Figure 19c; the stress-strain curves of failure process basically show a one stage sharp decline before failure, as there is no carbon fiber at the top layer delaying the failure start time, as shown in Figure 19a,b,d,e.

**Figure 20:**
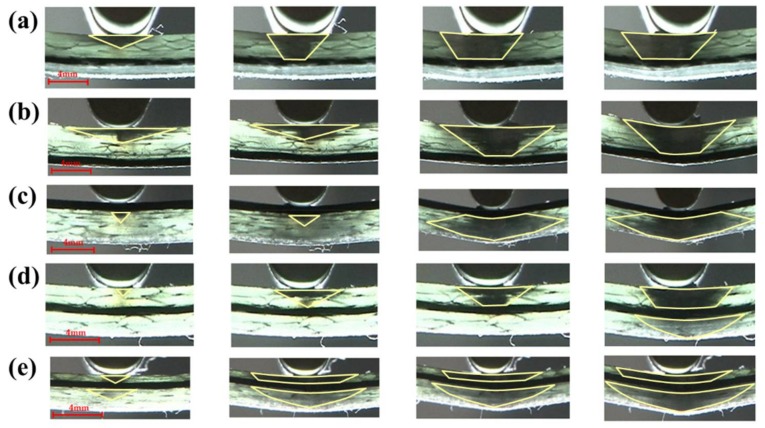
Flexure failure process of various interlayer stacking sequences with C/G = 1:4: (**a**) [G/G/G/C/G]; (**b**) [G/G/G/G/C]; (**c**) [C/G/G/G/G]; (**d**) [G/G/C/G/G]; (**e**) [G/C/G/G/G]. Note: the yellow zone indicates the failure zone of glass fiber.

As can be seen from the flexure failure process of interlayer hybrid composites with C/G = 1:4, shown in Figure 20, a diamond-shaped failure area appears, with carbon fiber distributed in the top layer, like [C/G/G/G/G] in Figure 20c. When there is no carbon fiber in the top layer, the failure area of the glass fiber layers above the carbon fiber presents an inverted trapezoid shape in Figure 20a,b, yet the failure area of the glass fiber layers below the carbon fiber presents an inverted triangular shape in Figure 20d,e.

### 3.3. Flexural Progressivefailure Modes of Intralayer Hybrid Composites

With regards to the intralayer hybrid composites, carbon fiber distributes in each layer, and since carbon fiber is light-proof, it is difficult to observe the bending damage zone with the light transmittance method. Therefore, the failure modes were analyzed mainly by the deflection-force curves and the sound fracture time-domain analysis.

**Figure 21:**
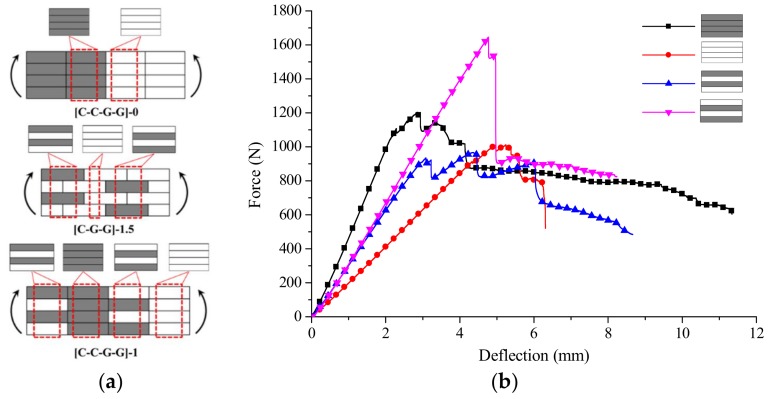
The relationship between the intralayer and interlayer structures. (**a**) Structure characteristics of intralayer hybrid composites; (**b**) Force-deflection curves of four structures from intralayer hybrid.

Intralayer hybrid composites could be considered as the mixed forms of four structures, inclusive of [C/C/C/C], [G/G/G/G], [C/G/C/G], [G/C/G/C]. For example, for the intralayer structure [C/C/G/G]-0 in Figure 21a, which only comprises [C/C/C/C], [G/G/G/G], for the structure [C/G/G]-1.5, which contains [C/G/C/G], [G/G/G/G], and [G/C/G/C], and so forth, [C/C/G/G]-1 consists of [C/G/C/G], [C/C/C/C], [G/C/G/C] and [G/G/G/G]. The failure modes of intralayer hybrid composites can be analyzed combined with these four structures, and their force-deflection curves are shown in Figure 21b.

**Figure 22:**
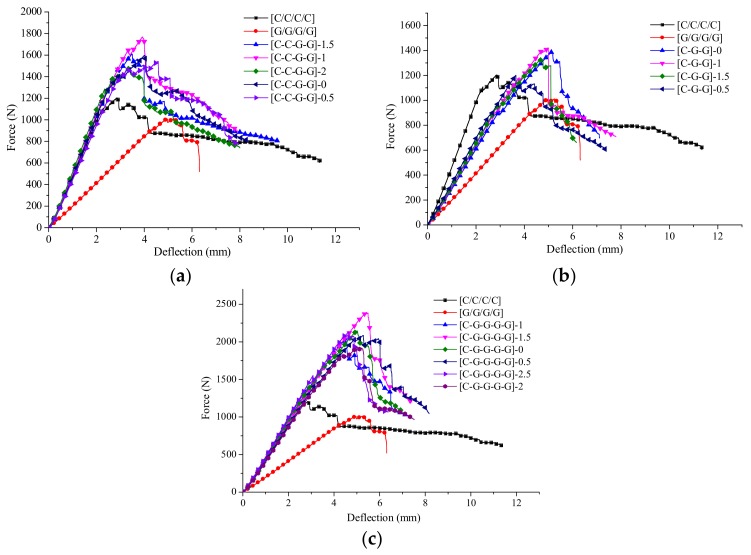
Force-deflection curves of various intralayer stacking sequences: (**a**) C/G = 2:2; (**b**) C/G = 1:2; (**c**) C/G = 1:4.

From the force-deflection curves of intralayer hybrid composites shown in Figure 22, there are various flexural failure modes of intralayer hybrid composites with different hybrid structures and mixed ratios. When the C/G hybrid ratio is 2:2, the bending force attenuation rate is the minimum, and the failure process is comparatively long. By contrast, as the C/G ratio is 1:4, the bending force attenuation rate is the maximum, and the failure time is short, the force attenuation rate with the C/G ratio 1:2 is between that of the former two cases. Therefore, the mechanical attenuation of intralayer hybrid composites increase evidently as glass fiber content increases, and conforms well to the rule of mixture.

The bending failure process for intralayer hybrid composites is more complex, featured with a multi-stage fluctuating drop shown in Figure 22. Since the intralayer hybrid contains carbon and glass fiber in each layer, carbon fiber in the top layer crushes quickly, and no longer bears the flexural force when the damage occurs; yet, glass fiber still assumes a certain amount of force leading to the downtrend falling sharply. Thus, after the first layer completely fails, the second layer starts to fail, which results in the deflection-force curve showing a multi-stage fluctuating decline due to the different failure order between carbon and glass fiber.

**Figure 23:**
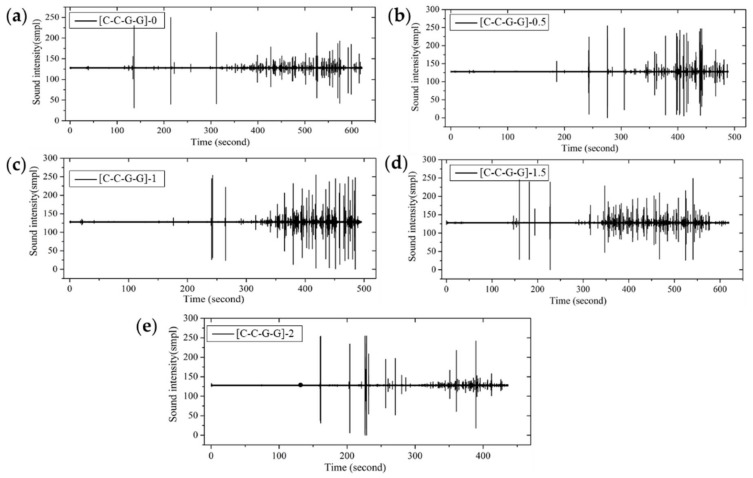
Flexure fracture sound intensity-time curve of various intralayer stacking sequences with C/G = 2:2: (**a**) [C-C-G-G]-0; (**b**) [C-C-G-G]-0.5; (**c**) [C-C-G-G]-1; (**d**) [C-C-G-G]-1.5; (**e**) [C-C-G-G]-2.

**Figure 24:**
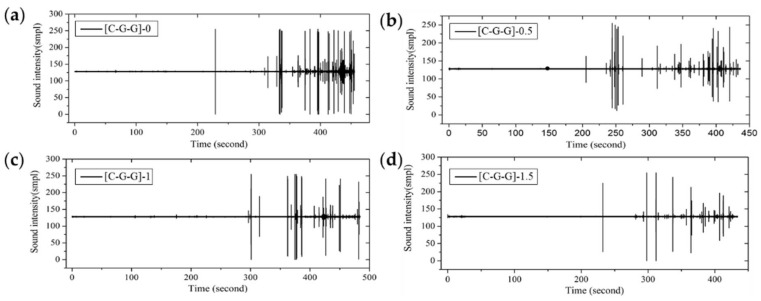
Flexure fracture sound intensity-time curve of various intralayer stacking sequences with C/G = 1:2: (**a**) [C-G-G]-0; (**b**) [C-G-G]-0.5; (**c**) [C-G-G]-1; (**d**) [C-G-G]-1.5.

**Figure 25:**
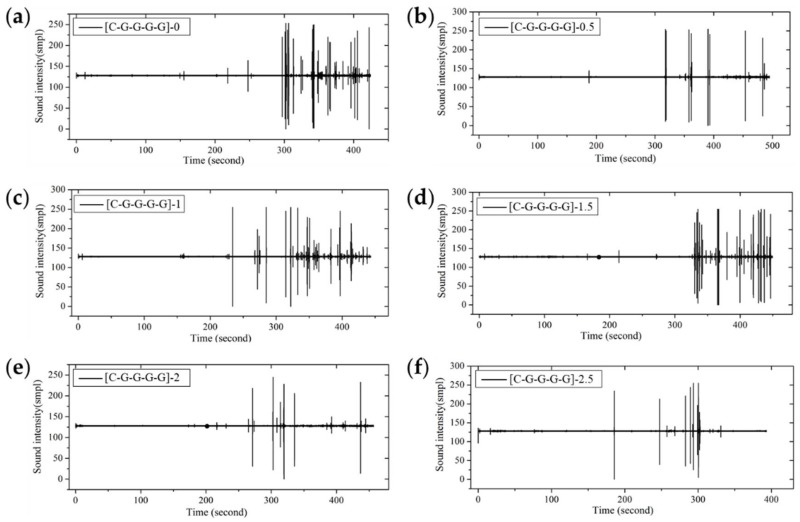
Flexure fracture sound intensity-time curve of various intralayer stacking sequences with C/G = 1:4: (**a**) [C-G-G-G-G]-0; (**b**) [C-G-G-G-G]-0.5; (**c**) [C-G-G-G-G]-1; (**d**) [C-G-G-G-G]-1.5; (**e**) [C-G-G-G-G]-2; (**f**) [C-G-G-G-G]-2.5.

From the fracture sound intensity time-domain plots shown in Figure 23, Figure 24 and Figure 25, as glass fiber content increases, the fracture failure of intralayer hybrid composites occurs late, due to the high fracture strain of glass fiber, the fracture sound release tends to be featured with a high intensity for low frequency. By contrast, with the increase of carbon fiber content, the failure time starts early, and there are two or three fracture sound releases for high intensity firstly, and then the release is featured with a low intensity and high frequency.

## 4. Conclusions

The flexural properties of interlayer and intralayer composites with various hybrid ratios and layup structures were tested in this work, and the bending behaviors and progressive failure modes were analyzed via the light transmittance method and the fracture sound release curves.

This study has shown that,

1. The bending force of pure carbon fiber composites shows a mild decline after the failure, and the failure fracture is featured with a low intensity and high frequency for a long time. The force decline of pure glass fiber composites appears more sharply, while the failure sound release is featured with a high intensity and low frequency for a short time. An inverted triangular failure zone appears for both carbon and glass fiber composites.
2. The progressive failure modes of interlayer hybrid composites are determined by the layup structure. As carbon fiber locates at the top surface for four C/G hybrid ratios of interlayer hybrid composites, the failure start point is early and the force/stress shows a multi-stage slightly fluctuating decline, the failure area exhibits a diamond shape. As carbon fiber distributes in the middle or bottom layers, and the failure start time is late, and the failure process exhibits a one-stage sharp force/stress drop, the fracture zone of glass fiber above the carbon layers presents an inverted trapezoid shape, while the fracture of glass fiber below the carbon layers exhibits an inverted triangular shape.
3. The mixed ratio plays a dominating role for intralayer hybrid composites in the bending failure, and intralayer hybrid composites could be considered as the mixed forms of four structures, including [C/C/C/C], [G/G/G/G], [C/G/C/G], [G/C/G/C]. As carbon fiber content increases, the failure time starts early and the fracture sound release is featured with a low intensity and high frequency for a long time, and the bending force shows a multi-stage fluctuating decline due to the different failure order of carbon and glass fiber. By contrast, as glass fiber content increases, the high strain of glass fiber delays the bending failure, and the fracture sound release exhibits a multi-stage sharp decline with a high amplitude and low frequency for a short time.

## Figures and Tables

**Table 1:** Constituent materials and selected properties.

Material	Tensile Strength (MPa)	Tensile Modulus (GPa)
CPIC ECT469L-2400 Glass Fiber	2366	78.7
TORAY T620SC-24K-50C Carbon Fiber	4175	234
SWANCOR 2511-1A/BS Epoxy Resin	73.5	3.1

**Table 2:** Specifications for hybrid fabric.

Fabric Type	Areal Density (g/m^2^)		Ratio of C/G
	Carbon Fiber	Glass Fiber	
Carbon	728.3	0	1:0
Glass	0	944.9	0:1
C-C-G-G	364.2	472.4	1:1
C-G-G	242.8	629.9	1:2
C-G-G-G-G	145.7	755.9	1:4

**Table 3:** Stacking configurations of interlayer hybrid structures.

C/G Hybrid Ratios	Stacking Sequences					
C:G = 1:1	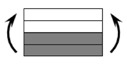	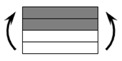	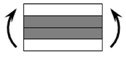	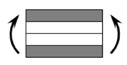	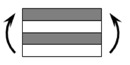	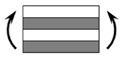
	[G/G/C/C]	[C/C/G/G]	[G/C/C/G]	[C/G/G/C]	[C/G/C/G]	[G/C/G/C]
C:G = 1:2	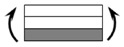	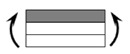	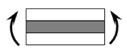			
	[G/G/C]	[C/G/G]	[G/C/G]			
C:G = 1:3	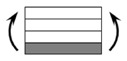	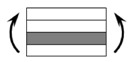	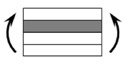	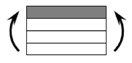		
	[G/G/G/C]	[G/G/C/G]	[G/C/G/G]	[C/G/G/G]		
C:G = 1:4	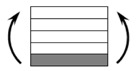	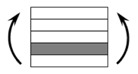	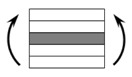	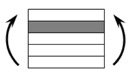	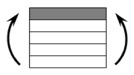	
	[G/G/G/G/C]	[G/G/G/C/G]	[G/G/C/G/G]	[G/C/G/G/G]	[C/G/G/G/G]	

**Table 4:** Stacking configurations of intralayer hybrid structures.

C/G Hybrid Ratios	Ply Sequences					
[C-C-G-G] C:G = 1:1	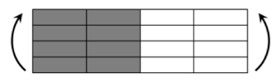		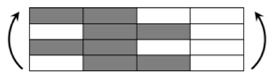		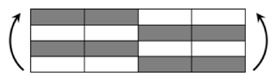	
	[C-C-G-G]-0		[C-C-G-G]-1		[C-C-G-G]-2	
C-G-G C:G = 1:2	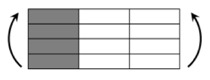	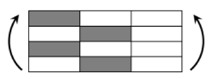		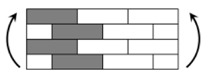		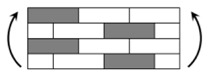
	[C-G-G]-0	[C-G-G]-1		[C-G-G]-0.5		[C-G-G]-1.5
C-G-G-G-G C:G = 1:4	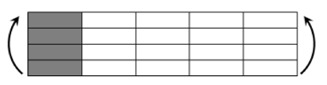		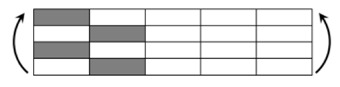		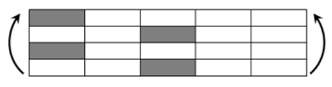	
	[C-G-G-G-G]-0		[C-G-G-G-G]-1		[C-G-G-G-G]-2	
	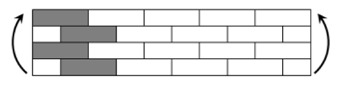		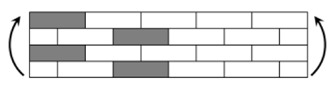		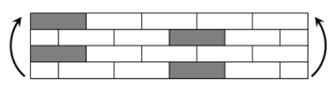	
	[C-G-G-G-G]-0.5		[C-G-G-G-G]-1.5		[C-G-G-G-G]-2.5	
